# IDO1 induced macrophage M1 polarization via ER stress-associated GRP78-XBP1 pathway to promote ulcerative colitis progression

**DOI:** 10.3389/fmed.2025.1524952

**Published:** 2025-04-30

**Authors:** Zijian Gao, Shuai Shao, Zhen Xu, Jiao Nie, Chenglin Li, Chao Du

**Affiliations:** ^1^Linyi People’s Hospital, Shandong Second Medical University, Linyi, China; ^2^Department of Gastroenterology, Linyi People’s Hospital, Shandong Second Medical University, Linyi, China; ^3^Department of Oncology, Linyi People’s Hospital, Shandong Second Medical University, Linyi, China; ^4^Department of Gastroenterology, Weihai Municipal Hospital, Shandong University, Weihai, China

**Keywords:** ulcerative colitis, macrophages, indoleamine 2,3-dioxygenase-1, GRP78, XBP1

## Abstract

Ulcerative colitis (UC) is a chronic inflammatory bowel disorder distinguished by alternating phases of remission and exacerbation. Restoring immune balance through the modulation of M1 macrophage polarization represents a potentially valuable therapeutic strategy for UC. Indoleamine 2,3-dioxygenase-1 (IDO1) has been shown to contribute to macrophage plasticity, but its role in the pathogenesis of UC via regulating M1 macrophage polarization has not been studied yet. For the clinical component, we analyzed IDO1 expression in UC using bioinformatics analysis of Gene Expression Omnibus (GEO) datasets and validated the result using western blotting of colonic tissues from new recruited UC patients. Colitis was induced in mice via dextran sulfate sodium (DSS) treatment and subsequently treated with oral administration of 1-methyl-DL-tryptophan (1-MT), an inhibitor of IDO1 pathway. The results indicated that IDO1 expression was significantly elevated in UC patients and correlated with M1 macrophage polarization observed in both human data and colitis mice. Furthermore, 1-MT markedly ameliorated DSS-induced weight loss, colonic shortening and disease severity via inhibiting IDO1 expression level, downregulating GRP78-XBP1 pathway and reducing M1 proportion. Notably, *in vitro* study revealed that overexpressing IDO1 in RAW264.7 cells induced macrophage M1 polarization with increased expression levels of GRP78 and XBP1, which was attenuated by 1-MT treatment. Additionally, the catalytic effect exerted by IDO1 overexpression on M1 polarization was neutralized by employing an inhibitor targeting the endoplasmic reticulum (ER) stress pathway. Thus, our findings suggest that IDO1 may promote UC progression by skewing macrophages towards M1 polarization through ER stress-associated GRP78-XBP1 pathway.

## 1 Introduction

Ulcerative colitis (UC) is an idiopathic, chronic inflammatory disease of the colonic mucosa with rising risk of deterioration in colorectal function, hospitalization, surgery, tumor development and other extrinsic complications (1–3). Although its etiology remains incompletely understood, intestinal immune modulation plays a critical role (4, 5). Macrophages are pivotal components of innate immunity and can exhibit either pro-inflammatory or anti-inflammatory functions, thereby facilitating the destruction or remodeling of local tissue (6). In the context of UC, macrophages are activated and recruited to the inflamed sites of the colon. Once present, they secrete various inflammatory mediators, such as inducible nitric oxide synthase (iNOS), interleukin-6 (IL-6), and tumor necrosis factor-alpha (TNF-α) (7, 8). Additionally, the excessive activation of M1-type macrophages leads to the disruption of the intestinal epithelial barrier (9). M1 macrophages also can interact with other immune cells, such as T cells and dendritic cells, modulating the immune response and perpetuating the inflammatory process (10). Therefore, the inhibition of M1 macrophage polarization might be a promising therapeutic approach for UC.

Indoleamine 2, 3-dioxygenase-1 (IDO1) is now considered as a well-established immune regulator in autoimmune diseases, chronic inflammation, and tumor immunity (11–14). In previous studies, it has been found that IDO1-mediated cytokine regulation stimulates macrophage activation in coronary atherosclerotic plaques, ultimately leading to increased thrombosis (15). Tryptophan depletion mediated by IDO1 can drive macrophage phenotypes towards a more pro-inflammatory state, thereby enhancing the secretion of inflammatory cytokines such as IL-6 (16, 17). Concurrently, pro-inflammatory cytokines secreted by M1 macrophages, including interferon-γ (IFN-γ), can upregulate IDO1 expression, establishing a feedback loop that further refines the immune response (18, 19). Furthermore, in autoimmune disorders such as rheumatoid arthritis, the aberrant activation of M1 macrophages and the dysregulated expression of IDO1 contribute to chronic inflammation and tissue damage (16). However, the expression pattern and functional outcome of IDO1 in UC via regulating macrophage polarization have not been elucidated yet.

In the context of UC, endoplasmic reticulum (ER) stress plays a pivotal role in the pathogenesis (20, 21). GRP78, a crucial molecular chaperone within ER, is one of the key markers for ER stress (22). During ER stress, the transmembrane proteins dissociate from GRP78, activating their endonuclease function to excise a 26-nucleotide intron from X-box binding protein 1 (XBP1) mRNA. The spliced XBP1 mRNA (XBP1s) is subsequently translated into the active transcription factor XBP1s, which enhances the expression of various target genes involved in the unfolded protein response (UPR) to mitigate ER stress and intestinal inflammation (23, 24). Recent research also underscores a significant correlation between ER stress and M1 polarization, as ER stress triggers intracellular signaling pathways that modulate transcriptional programs governing macrophage polarization (25). The UPR pathway activated by GRP78, such as the GRP78-XBP1 pathway regulates inflammatory responses related to M1 polarization (26). However, there is currently lack of research that elucidates the specific mechanisms by which ER stress-mediated macrophage M1 polarization contributes to UC. Moreover, IDO1 is likely to facilitate the pathological progression of UC through its involvement in the pathway of ER stress-induced macrophage M1 polarization.

In this study, we explored the mechanism of IDO1 acting on macrophage M1 polarization in UC by bioinformatic analysis UC-GEO datasets and western blotting analysis of IDO1 in newly recruited UC patients. Subsequently, DSS-induced colitis was used to measure the level of IDO1, GRP78, XBP1, and M1 marker iNOS correlated with disease activity, while 1MT treatment ameliorated DSS-induced colitis by downregulating IDO1-GRP78-XBP1 pathway. Further, LPS + IFN-γ-induced M1 phenotype with overexpression of IDO1 in RAW264.7 cell model was used. We validated our findings that IDO1 prompts macrophage polarization towards M1 via ER stress-associated GRP78-XBP1 pathway, thereby facilitating the progression of UC.

## 2 Materials and methods

### 2.1 Patient specimens

Four Gene Expression Database (GEO) datasets of patients with active UC and healthy controls were included in our study. The keywords utilized for the search include “*Homo sapiens*,” “ulcerative colitis,” “active’, “healthy control” and “mucosal biopsy.” Spain cohort GSE38713 (27), USA cohort GSE92415 (28) and Belgium cohorts GSE75214 (29) and GSE73661 (30) were used in this study (Table 1).

**Table 1:** Datasets utilized for analysis.

Dataset	Gene number	Platform	Case samples	Control samples
GSE38713	22,854	GPL570	15	13
GSE75214	23,298	GPL6244	97	11
GSE73661	23,320	GPL6244	23	12
GSE92415	25,293	GPL13158	87	21

The clinical tissue samples of 10 UC patients diagnosed according to ECCO guidelines for IBD (31) and 10 controls were obtained from Linyi People’s Hospital. Participants in the control group were selected from patients undergoing colonoscopy for polyps and cancer surveillance. Subjects were excluded if they had undergone major abdominal surgery; or had a history of malignant neoplasms. Colonic tissue samples from patients were taken under colonoscopy. This study was approved by the Medical Ethics Committee of Linyi People’s Hospital (YX200563).

### 2.2 Evaluation of M1 macrophage proportion in retrieved gene expression datasets by CIBERSORTx algorithm

We retrieved the datasets GSE38713, GSE92415, GSE75214, and GSE73661 from the GEO database and the CIBERSORTx algorithm was utilized to assess M1 macrophage infiltration in UC patients from the four GEO cohorts (32, 33). The transcriptomic dataset was uploaded as a mixed file to the CIBERSORTx web portal,^1^ and the LM22 signature matrix was utilized in the deconvolution analysis to define cell populations. The LM22 Signature Matrix, provided by CIBERSORT, is a reference database for analyzing the composition of immune cells in complex tissues (34). Combined with the CIBERSORT algorithm, it enables the deconvolution of bulk gene expression data to quantify immune cell subsets to support disease research and clinical prediction. The data output from CIBERSORTx was analyzed using the R software.

### 2.3 Correlation analysis between common differentially expressed genes and M1 macrophage proportion across various datasets

The extracted gene expression data from the four GEO cohorts were processed using the “limma” package in R software (version 4.4.1) with adjusted *p* < 0.05 and |logFC| >3 for identifying differentially expressed genes (DEGs) across varying conditions. Furthermore, we employed the “ggvenn” package to construct a Venn diagram for identifying the common DEGs across the four datasets. Additionally, we conducted a correlation analysis between these common DEGs and M1 macrophage proportion in each dataset using the “corrplot” package.

### 2.4 Gene Set Variation Analysis

To gain insights into the biological functions of extracted gene expression data in the context of Gene Ontology (GO) analysis they are involved in, the gene expression data in GSE38713, GSE92415, GSE75214, and GSE73661 were subjected to GO-cellular component (CC) pathway analysis using the Gene Set Variation analysis (GSVA) package, which is a non-parametric and unsupervised method for evaluating gene enrichment in transcriptomes. By providing a comprehensive score for interesting gene sets, GSVA converts genetic variation into pathway-level changes and further determines the biological functions of samples. The GSVA data was then processed using the “limma” package to identify the top CC pathways.

### 2.5 Animal experiments

Female C57BL/6 mice aged 6 to 8 weeks were purchased from JEKAIYER (Jinan, China). The experimental protocol was strictly in accordance with the ethical regulations of the Animal Care and Use Committee of Linyi People’s hospital. Animals were maintained at a temperature of 23 ± 2°C and a humidity of 55 ± 10% in a specific pathogen-free environment with a 12 h light/dark cycle and were provided free access to standard laboratory diet and water.

After acclimatization for at least 1 week, the mice were randomly divided into 5 groups (*n* = 5/each group): control group, DSS-Day2 group, DSS-Day7 group, DSS + 1-MT (2 mg/mL)-Day7 group and 1-MT (2 mg/mL) group. As previously described (35), colitis was induced by 3.5% DSS dissolved in drinking water for 7 consecutive days, while control mice drank the same volume of distilled water. 1-MT was dissolved in sweet-tasting weakly acidic drinking water at a dose of 2 mg/mL (36, 37) and given to mice daily, alone or with DSS, for 7 days. Body weights were recorded daily. Colitis severity was evaluated by the disease activity index (DAI) based on weight loss, stool bleeding and stool consistency (38, 39). Fecal specimens were systematically collected during the circadian morning active phase [Zeitgeber Time (ZT) 3–5]. Feces were immediately frozen after collection and stored in a vacuum sealed environment at −80°C, and unified detection was performed after the end of the experiment. After animals in each group were sacrificed, colons were measured and processed for histopathological studies (40), western blotting and enzyme-linked immunosorbent (ELISA) assay. This study was approved by the Science and Technology Ethics Committee of Linyi People’s Hospital (202410-A-002).

### 2.6 Cell culture, treatment and transfection

RAW 264.7 cells were cultured in Dulbecco Modified Eagle medium containing 10% (volume/volume) fetal bovine serum, 100 U/mL penicillin and 100 U/mL streptomycin (all from Gibco, United States) at 37°C and 5% CO_2_ in a plastic disposable cell culture flask (Corning, United States). To investigate the effect of IDO1 on the polarization of M1 macrophages, cells were divided into eight groups: control group, 6 h model group (LPS 20 μg/mL + IFN-γ 4 μg/mL), 24 h model group (LPS 20 μg/mL + IFN-γ 4 μg/mL), 1-MT (100 μM) + LPS-IFN-γ group, 1-MT (100 μM) group, IDO1-overexpression group, IDO1-overexpression + LPS-IFN-γ group and IDO1-overexpression + 1-MT + LPS-IFN-γ group. Cells were seeded at a density of 2 × 10^5^ cells/mL in 6-well plates (2 mL/well) and allowed to adhere for 24 h in a 37°C, 5% CO₂ incubator until reaching 70–80% confluency. For polarization induction, culture medium was replaced with fresh complete medium containing 20 μg/mL LPS and 4 μg/mL IFN-γ. Prior to stimulation, cells were gently washed twice with prewarmed PBS to remove residual cytokines. The IDO1 inhibitor 1-MT was dissolved in dimethylsulfoxide (DMSO; Sigma-Aldrich) prior to administration and cells were pretreated with 1-MT for 6 h prior to induction with LPS + IFN-γ. Three independent replications were performed for all the experiments. Upon reaching the designated treatment time points, cells from each experimental group were immediately harvested. RNA and protein were subsequently isolated using TRIzol-based dual extraction protocols, aliquoted into sterile cryovials, and cryopreserved at −80°C under vacuum-sealed conditions to ensure biomolecular stability for downstream analyses.

Overexpression plasmids were transfected into cells with E-trans TM (Genechem) according to the manufacturer’s protocol. Plasmids were synthesized by Genechem (Shanghai, China). Vector name and element order is listed in Table 2.

**Table 2:** Real-time PCR primers (F: forward primer; R: reverse primer) and vector element order.

Name	Sequence (5′–3′)/vector element order
GAPDH	F: TCGGGCCACGCTAATCTCAT
	R: ACGGCCAAATCCGTTCACA
IL-6	F: GCCTTCTTGGGACTGATGCT
	R: TGCCATTGCACAACTCTTTTC
TNF-α	F: CCCAAAGGGATGAGAAGTTCC
	R: GCTACAGGCTTGTCACTCGAA
GRP78	F: GAACACTGTGGTACCCACCAAG
	R: TCCAGTCAGATCAAATGTACCCAGA
XBP1s	F: GAGCAGCAAGTGGTGGATTTG
	R: CGTGTTCTTAACTCCTGGTTCTCA
IDO1	F: GCCTCCTATTCTGTCTTATGCAG
	R: ATACAGTGGGGATTGCTTTGATT
GV657	CMV enhancer-MCS-3flag-polyA-EF1A-zsGreen-sv40-puromycin

### 2.7 Quantitative real-time PCR and RNA sequencing

RAW264.7 cells were plated in 6-cm culture dishes to achieve 80% confluence. Total RNA was extracted using Trizol reagent (Thermo Fisher Scientific). The mRNA levels of IDO1, inflammatory cytokines and iNOS were evaluated by reverse transcription (RT) with Moloney murine leukemia virus (M-MLV) reverse transcriptase (Thermo Fisher), followed by conventional quantitative PCR (qPCR) with SYBR Green Pro Taq HS (Agbio, Hunan, China). The qPCR was performed on an ABI 7500 real-time PCR system (Thermo Fisher). GAPDH was used as an internal control. The oligonucleotide sequences of primers used are listed in Table 2. The PCR amplification conditions were as follows: predenaturation at 95°C for 30 s, followed by 40 cycles at 95°C for 5 s and 60°C for 35 s, and then the primers were unchained at 60°C for 1 min and 95°C for 15 s. Relative mRNA expression was calculated using the $2^{-\mathrm{\Delta \Delta CT}}$ method.

The total RNA of the control group, LPS-IFN-γ group and IDO1-overexpression + LPS-IFN-γ group was extracted with Trizol reagent (Invitrogen, Carlsbad, United States). Total RNA concentration and purity were measured using an ND-1000 NanoDrop spectrophotometer (NanoDrop Technologies, Wilmington, United States). Quality control and deep sequencing are conducted by Major Bio (Shanghai, China). Utilizing the MajorBio Cloud platform (Shanghai, China), Gene Ontology (GO) analysis and Kyoto Encyclopedia of Genes and Genomes (KEGG) pathway analysis were performed on the genes derived from sequencing with the top six pathways visualized using MajorBio Cloud platform.

### 2.8 Enzyme-linked immunosorbent assay

Colon tissue of colitis mice was homogenized in PBS buffer (pH 7.4) and centrifuged at 14,000 g and 4°C for 30 min. Supernatants were collected and stored at −80°C for further analysis. The protein concentration was measured using a BCA protein assay kit (Beyotime, Jiangsu, China), and the level of iNOS, IL-6, TNF-α was detected by ELISA kit.

### 2.9 Protein extraction and western blot analysis

Total protein extracts were obtained from colon tissues or RAW264.7 in ice-cold RIPA lysis buffer (Beyotime) with phosphatase inhibitor mixture and protease inhibitor (Thermo Fisher). Using the BCA protein assay kit (Beyotime) quantitative concentration. The extracted proteins were separated by SDS-PAGE and transferred onto 0.22 μm polyvinylidene difluoride membranes (Millipore, MA, United States). Membranes were blocked with 5% non-fat powdered milk for 2 h at room temperature (25–30°C), and then incubated separately with rabbit antibodies against β-tubulin (1:2,000; Cat No. 2128S; CST), XBP1s (1:1,000; Cat No. 12782S; CST), iNOS (1:500; Cat No. PA1-036; Thermo Fisher) and rat antibodies against GRP78 (1:200; Cat No. sc-13539; Santa Cruz Biotechnology), IDO1 (1:200; Cat No. sc-53978; Santa Cruz Biotechnology) in TBST (Tris-buffered saline, 0.1% Tween 20) buffer at 4°C overnight. Subsequently, membranes were incubated with HRP-conjugated goat anti-rabbit IgG or HRP-conjugated goat anti-mouse IgG secondary antibody (Thermo Fisher Scientific) for 1.5 h at room temperature. The protein was visualized by BeyoECL Star (Beyotime) using chemiluminescence and quantified with Image J software.

### 2.10 Reagents

1-methyl-DL-tryptophan (1-MT) and Lipopolysaccharides (LPS) were purchased from Sigma Chemical Co (St. Louis, MO, United States). IFN-γ was purchased from Peprotech (Rocky Hill, NJ, United States) and the reconstitution was done in 1% BSA in phosphate buffer saline (PBS) at a concentration of 1,000 μg/mL and stored at −80°C. 4-Phenylbutyric acid (4-PBA) was purchased from APEXBIO (China) and dissolved in sterile, enzyme-free water and stored in a refrigerator at −80°C. Antibody name (ratio, product number, manufacturer): β-tubulin (1:2,000; Cat No. 2128S; CST), XBP1s (1:1,000; Cat No. 12782S; CST), iNOS (1:500; Cat No. PA1-036; Thermo Fisher) and rat antibodies against GRP78 (1:200; Cat No. sc-13539; Santa Cruz Biotechnology), IDO1 (1:200; Cat No. sc-53978; Santa Cruz Biotechnology).

### 2.11 Statistical analysis

Results are presented as mean ± standard error of mean (SEM). Statistical analysis was performed using one-way analysis of variance with Tukey’s multiple comparison test to analyze the significance among multiple groups, and an unpaired Student’s *t*-test was used to analyze the significance between the two groups. Differences were considered to be statistically significant if *p* < 0.05. No exclusion criteria were incorporated into the design of the experiments for this study.

## 3 Results

### 3.1 IDO1-GRP78-XBP1 demonstrates a strong correlation with M1 macrophages during the UC acute phase

**Figure 1:**
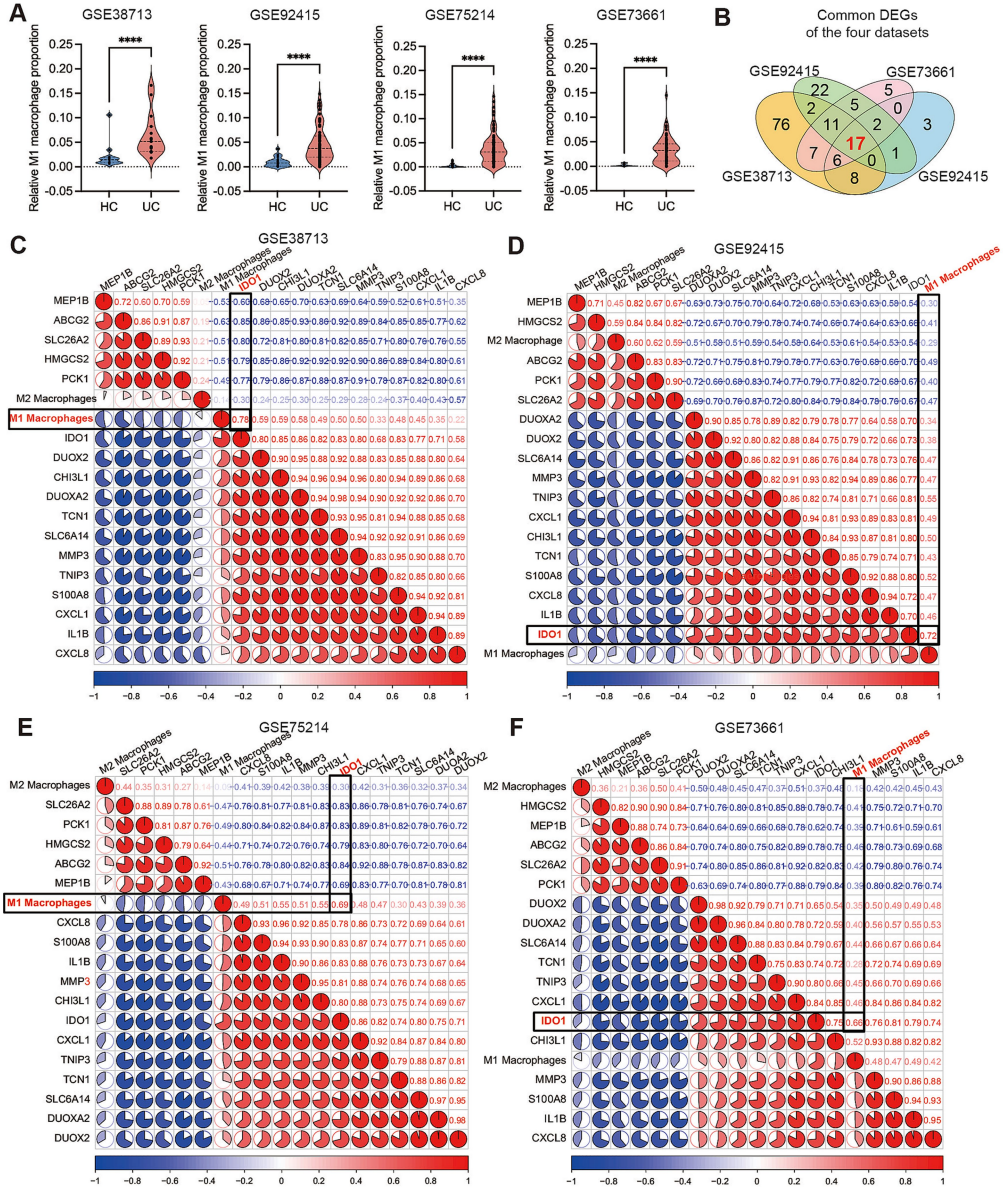
IDO1 demonstrates a strong correlation with M1 macrophages during the UC acute phase. **(A)** The small violin plot in the figure shows the difference in immune infiltration of M1 macrophages between normal individuals and ulcerative colitis patients. Blue represents the healthy control group, and red represents the ulcerative colitis group. **(B)** The Venn diagram analysis of differentially expressed genes (determined by DEG algorithm) across four datasets. **(C–F)** The co-expression pattern between macrophages and 17 key DEGs. Red: positive correlation; blue: negative correlation. ^****^p < 0.0001.

Macrophages play a crucial role in chronic inflammation and pathological processes. Research has demonstrated that the number of M1 macrophages significantly increases during active UC, indicating their involvement in the pathogenesis of UC (7, 41). Consequently, we employed the CIBERSORTx web portal to assess the differences in M1 macrophage proportion between the UC group and healthy control group across four cohorts obtained from the GEO database. The results showed that the infiltration of M1 macrophages was significantly increased UC patients in the active phase, and the results of the four cohorts were consistent (Figure 1A). To further elucidate the upstream regulatory mechanisms governing macrophage polarization in UC, we performed DEG analysis on each selected cohort (adjusted *p* < 0.05, |logFC| >3) and conducted an intersection analysis of the DEGs identified across all cohorts, revealing that a total of 17 common DEGs exhibited significant differences in the four cohorts (Figure 1B). Subsequently, we employed the “corrplot” package to conduct a correlation analysis between the 17 selected common DEGs and M1 macrophage proportion across the four cohorts. The results indicated that IDO1 exhibited the strongest correlation with M1 macrophages in all cohorts (Figures 1C–F). In parallel, our analysis of publicly available single-cell RNA sequencing dataset (42) revealed that compared with healthy controls (HC), M1 macrophages predominantly localized in tissues from patients with acute-phase UC and Crohn’s disease (Supplementary Figure 1). This study further demonstrated that IDO1 overexpression was principally concentrated within these M1 macrophage populations (Supplementary Figure 1). Subsequent co-expression analysis additionally identified possible concurrent enrichment of both GRP78 and XBP1s in highly-IDO1-expressing M1 macrophages (Supplementary Figure 1).

**Figure 2:**
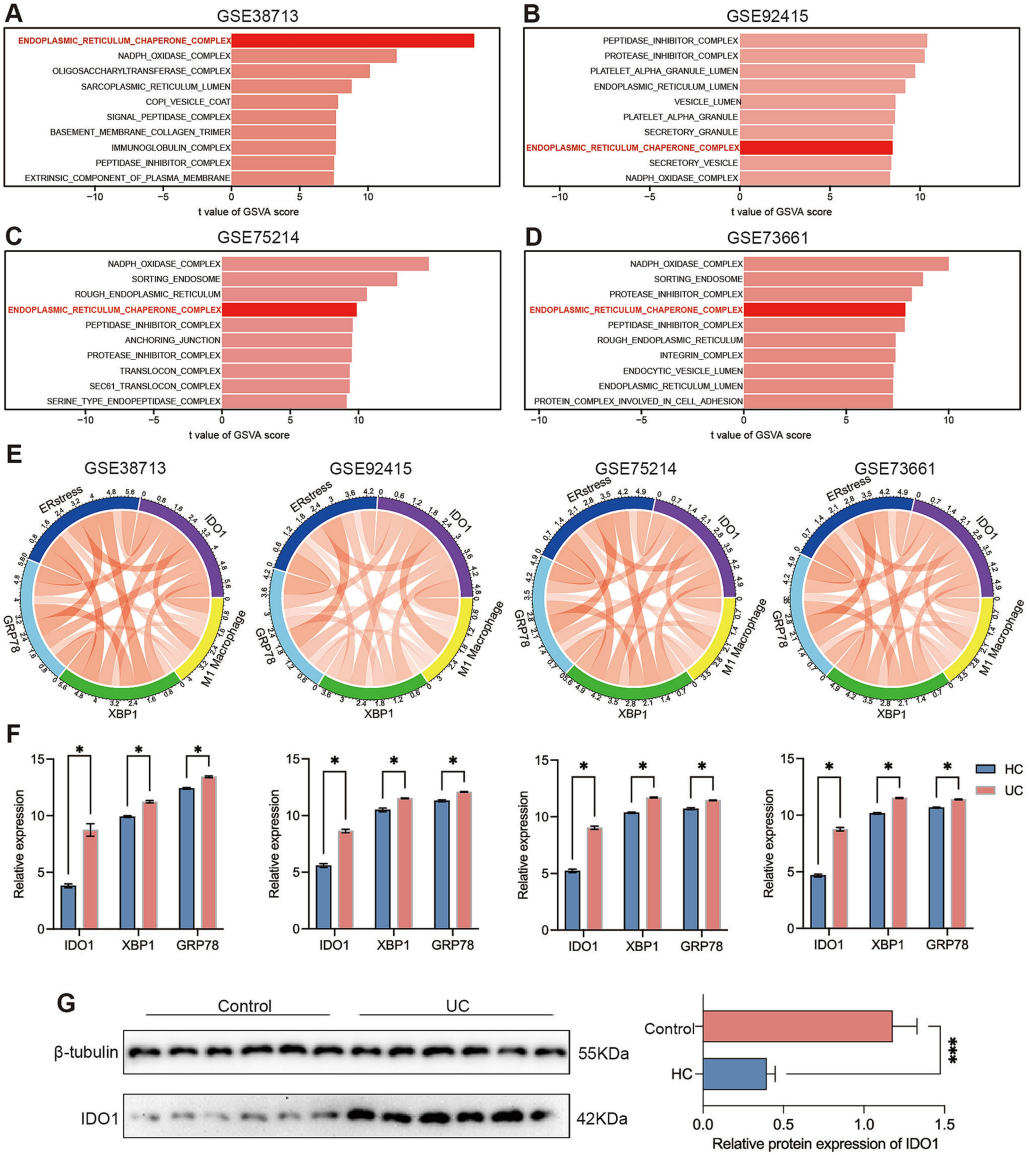
IDO1 modulates macrophage M1 polarization and is associated with endoplasmic reticulum stress. **(A–D)** The GSVA algorithm was employed to generate comprehensive scores for four gene sets in order to assess the cellular components that are pivotal in the progression of ulcerative colitis, including the endoplasmic reticulum chaperone complex, translocon complex, NAPDH oxidase complex. **(E)** The correlation of GRP78, XBP1, IDO1, ER stress, and M1 macrophage was identified by corrplot package. The red curve represents positive correlation. **(F)** The relative expression quantities of IDO1, GRP78, and XBP1s between the healthy control group and the UC group in each dataset. **(G)** The protein level of IDO1 in the colon tissue of UC patients. ^*^p < 0.05 and ^***^p < 0.001.

To further investigate the potential mechanism of IDO1 in macrophage polarization in the progression of UC, we used the GSVA algorithm to perform differential analysis of the cellular component (CC) terms within the gene expression profiles across all cohorts. The results indicated that the endoplasmic reticulum chaperone complex exhibited significant differential expression in the four cohorts (Figures 2A–D). The Chord Diagram analysis using the “circlize” package indicated a strong significant positive correlation among GRP78, XBP1, IDO1, ER stress, and M1 macrophages (Figure 2E). The expression levels of IDO1, GRP78 and XBP1 were significantly elevated during the acute colitis phase of UC in the four GEO cohorts (Figure 2F). Furthermore, we confirmed the increased expression of IDO1 in UC by performing western blotting analysis in newly recruited UC patients (Figure 2G).

### 3.2 Inhibition of IDO1 leads to relief of colitis under DSS challenge

**Figure 3:**
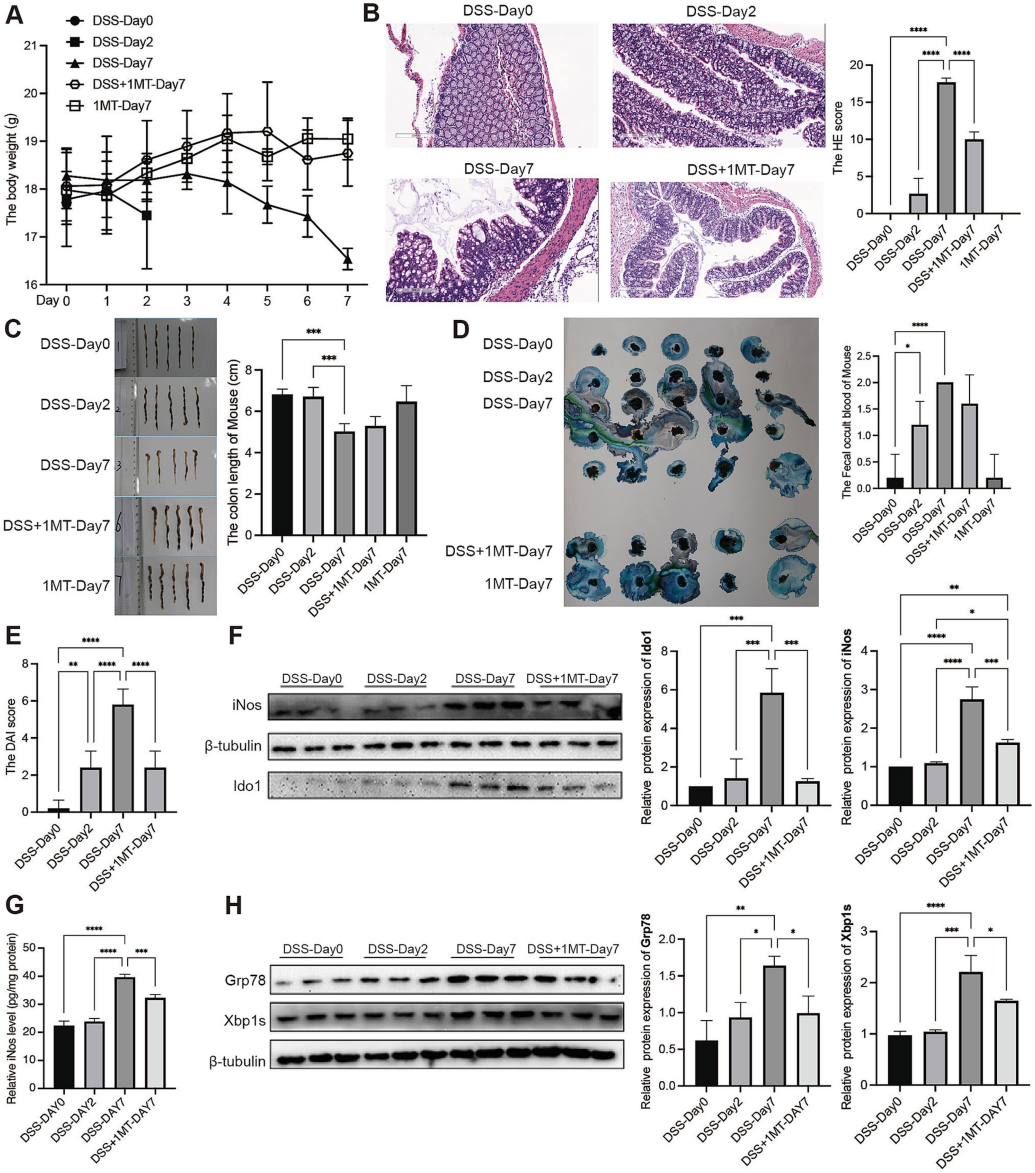
IDO1 regulates the XBP1-GRP78 pathway to boost mouse intestinal macrophage polarization and UC development. **(A)** Daily body weight change of mouse. **(B)** Haematoxylin-eosin stained sections of colon tissue observed under microscope (scale bar, 200 μm). **(C)** Length of mice colon. **(D)** The fecal occult blood of mouse. **(E)** The DAI score of mouse. **(F)** The protein level of iNOS and IDO1 in mouse colon tissue. **(G)** The level of iNOS in mouse colon tissue. **(H)** The protein expression level of GRP78 and XBP1s in mouse colon tissue. ^*^p < 0.05, ^**^p < 0.01, ^***^p < 0.001, and ^****^p < 0.0001.

To investigate the impact of IDO1 on UC development, we used DSS-induced colitis mice and inhibited IDO1 using 1-MT (43). After DSS administration, we observed severe colitis at day 7 characterized by greater weight loss, higher HE score, shorter colon length and fecal bleeding, with significantly higher DAI scores as compared to control group and day 2 after colitis (Figures 3A–E). However, administration of 1-MT significantly (2 mg/mL) ameliorated DSS-induced colitis in mice, reversed weight loss (Figure 3A), decreased HE score (Figure 3B) and decreased DAI score (Figure 3E). These findings collectively revealed that inhibition of IDO1 exhibited a strong therapeutic effect in ameliorating DSS-induced colitis.

Western blotting analysis indicated that IDO1 and iNOS, a molecular marker for M1-polarized macrophage, was significantly increased after induction of colitis (Figure 3F). Also, ELISA results showed that markers of M1 macrophages (iNOS, IL-6, TNF-α) in colon tissue increased significantly during the colitis stage (Figure 3G; Supplementary Figure 2). However, the expression of both M1 makers and IDO1 was significantly downregulated after treatment with 1-MT in DSS-induced colitis mice (Figures 3F,G; Supplementary Figure 2).

To further verify that IDO1 might cause M1 macrophage polarization via the GRP78-XBP1 pathway to promote colitis development, we detected the expression of GRP78 and XBP1s in colitis mice. The study findings suggested that the expression levels of GRP78 and XBP1s were significantly increased after colitis induction, which was consistent with the results in human datasets. However, when IDO1 was inhibited by 1-MT, the colitis severity was significantly ameliorated with a marked decrease in the expression levels of both GRP78 and XBP1s (Figure 3H).

### 3.3 IDO1 regulated M1 macrophage polarization by modulating GRP78-XBP1 pathway in RAW264.7 cells

**Figure 4:**
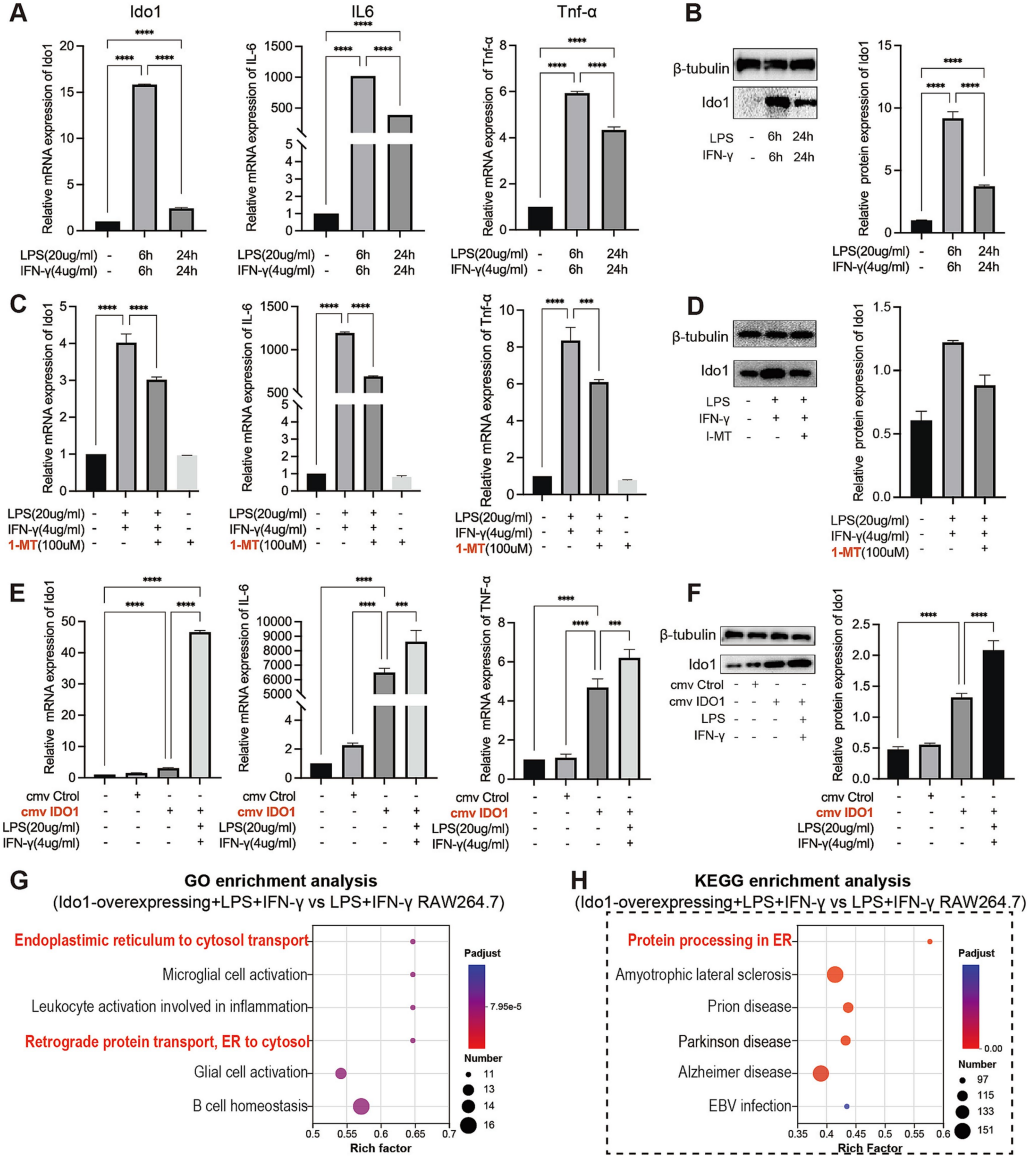
IDO1 promotes the inflammatory response and M1 polarization of RAW264.7 cells. **(A,C,E)** qPCR analysis of IDO1, IL-6, and TNF-α relative mRNA expression in RAW264.7 cells under various treatment conditions. **(B,D,F)** The relative protein expression of IDO1 in RAW264.7 cells under various treatment conditions. **(G,H)** The bubble plot showing the most enriched GO terms **(G)** and KEGG pathways **(H)** of DEGs. The screening criteria for significant enriched biological processes and pathways were adjusted *p* < 0.05. ^***^p < 0.001 and ^****^p < 0.0001.

To elucidate the role of IDO1 in the regulation of M1 macrophage polarization *in vitro*, we used LPS + IFN-γ to create the transition towards M1 phenotype in RAW264.7 cells. We observed a significant increase in the mRNA and protein levels of IDO1 and elevated mRNA levels of inflammatory factors after LPS + IFN-γ induction for 6 h and 24 h, with the most significant increase at 6 h (Figures 4A,B). Concurrently, a 6 h pretreatment with 1-MT (100 μM) effectively reversed the expression level of IDO1 and production of IL-6 and TNF-α induced by LPS + IFN-γ (Figures 4C,D). However, overexpression of IDO1 induced the production of IL-6 and TNF-α, and aggravated the production of those proinflammatory cytokines induced by LPS + IFN-γ (Figures 4E,F).

**Figure 5:**
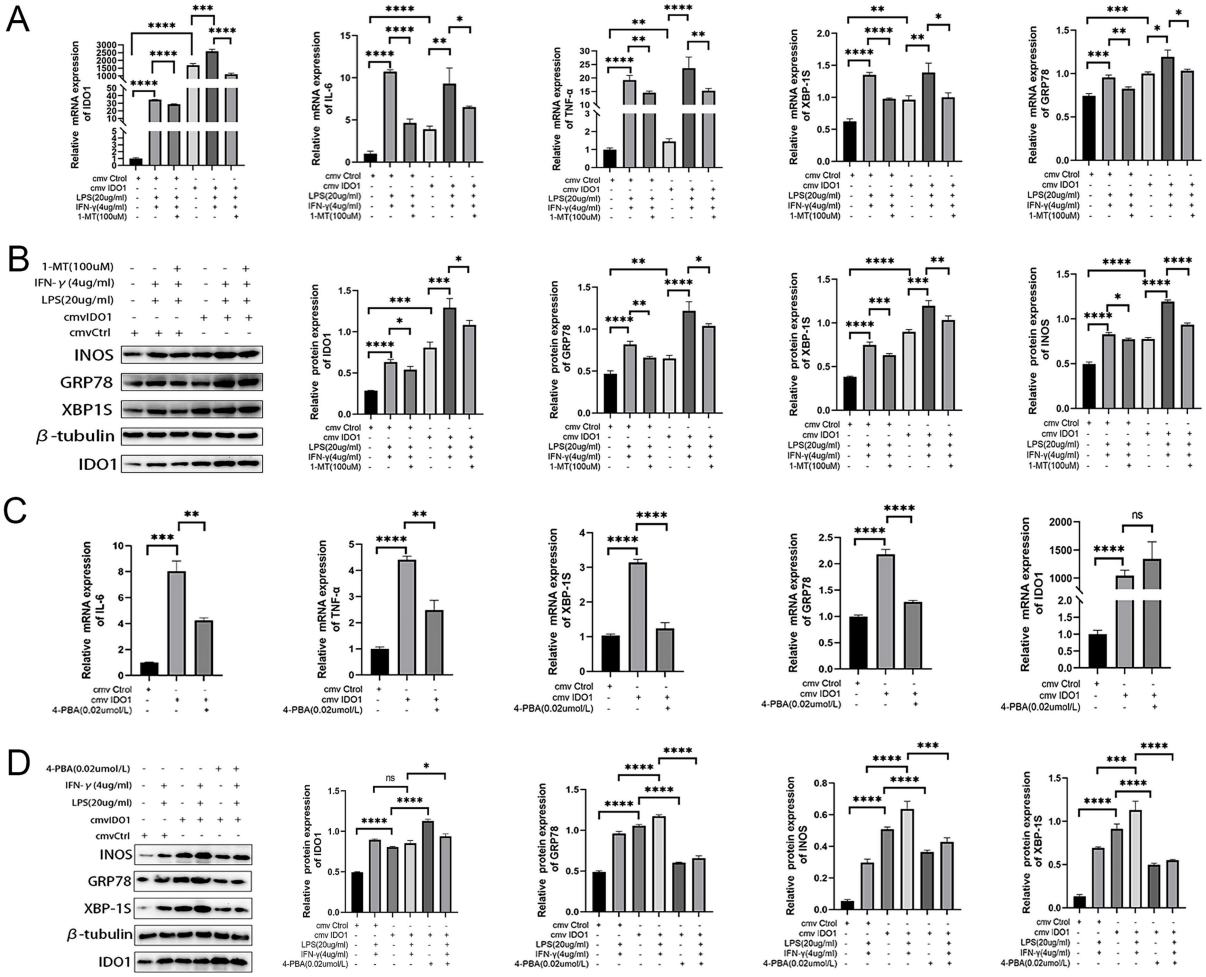
The M1 polarization of RAW264.7 cells mediated by IDO1 can be inhibited by 4-PBA. **(A,C)** The mRNA expressions of IDO1, GRP78, XBP1s, TNF-α, and IL-6. **(B,D)** The protein expression level of IDO1, markers of ER stress pathways (GRP78 and XBP1s) and M1 macrophage marker (iNOS). ns: no significant, ^*^p < 0.05, ^**^p < 0.01, ^***^p < 0.001, and ^****^p < 0.0001.

Subsequently, RNA sequencing was performed to investigate GO and KEGG enrichment pathways involved in overexpression of IDO1 in RAW264.7 cells. The results showed that the gene expression data were enriched in endoplasmic reticulum to cytosol transport pathway (GO analysis, Figure 4G) and protein processing in ER pathway (KEGG analysis, Figure 4H) in IDO1-overexpressing LPS + IFN-γ RAW264.7 cells as compared to LPS + IFN-γ RAW264.7 cells. Also, the mRNA level (Figure 5A) and protein level (Figure 5B) of GRP78 and XBP1s were significantly increased in IDO1-overexpression RAW264.7 cells and were more significantly upregulated in IDO1-overexpressing LPS + IFN-γ RAW264.7 cells; while, pretreatment with 1-MT reversed the increase of GRP78 and XBP1s (Figures 5A,B).

To further validate the role of IDO1 in macrophages, the expression level of iNOS was detected and it was revealed that overexpression of IDO1 increased the level of iNOS and more significantly upregulated iNOS expression in IDO1-overexpressing LPS + IFN-γ RAW264.7 cell. The results above demonstrated that IDO1 might induce the transition towards M1 macrophage phenotype via ER stress-associated GRP78-XBP1 pathways.

### 3.4 The M1 polarization of RAW264.7 cells mediated by IDO1 can be inhibited by 4-PBA

To further investigate whether IDO1 regulates the M1 polarization of RAW264.7 cells via the ER stress pathway, we employed 4-PBA to inhibit ER stress signaling in RAW264.7 cells (44, 45). The data indicated that although the mRNA level of IDO1 was not significantly decreased with 4-PBA treatment, the production of IL-6 and TNF-α as well as the mRNA level of GRP78 and XBP1s were significantly reduced (Figure 5C). In LPS + IFN-γ-induced RAW264.7 cells, administration of 4-PBA significantly down-regulated iNOS expression level and decreased the level of GRP78 and XBP1s (Figure 5D).

## 4 Discussion

Despite substantial advancements in understanding the pathogenesis of UC in recent years, the interactions between intestinal immune cells and UC, along with their underlying molecular mechanisms, remain inadequately defined (46, 47). Dysregulation of immune function within the intestinal microenvironment mediated by macrophages is a recognized contributor to UC pathology (3, 7). Previous studies have demonstrated that IDO1, a pivotal immune regulator, plays an essential role in macrophage polarization and various diseases, particularly in autoimmune disorders (11–15). ER stress signifies a disruption of cellular homeostasis and is capable of regulating M1 polarization of macrophages and alleviating intestinal inflammation associated with UC (23–26). Therefore, macrophage polarization mediated by IDO1 might be realized via ER stress and serves as a potential therapeutic target for modulating the immune response in UC.

In the preliminary investigation, bioinformatics analysis of the GEO datasets revealed a significant increase in M1 macrophage proportion in UC patients, with the strongest correlation with IDO1. Additionally, CC pathway analysis indicated that there was notable variation in ER chaperone complex composition pathway in UC patients. Given that the GRP78-XBP1 pathway is crucial for ER stress and might be closely linked to M1 macrophage polarization (20, 21, 25, 48), we selected GRP78 and XBP1 as markers for further research. Correlation analysis showed a positive relationship among IDO1, GRP78, XBP1, and ER stress and M1 macrophages in UC-GEO cohorts. Furthermore, expression levels of IDO1, GRP78, and XBP1 were significantly elevated in UC patients. Western blotting analysis of colon tissue from newly recruited UC patients confirmed significant upregulation of IDO1. Therefore, we propose that IDO1 may modulate M1 macrophage polarization and exacerbate intestinal inflammation via ER stress-associated GRP78-XBP1 pathway.

However, the bioinformatics and clinical data presented here only demonstrate an association between IDO1 and ER stress as well as M1 macrophage polarization in UC; they do not provide direct evidence that IDO1 regulates macrophage polarization to promote UC via the GRP78-XBP1 pathway. To further validate our hypothesis, we established a DSS-induced colitis model and evaluated the effects of IDO1 inhibition through oral administration of 1-MT. The results indicated that 1-MT-mediated inhibition of IDO1 alleviated colitis severity. Following suppression of IDO1 expression, levels of GRP78, XBP1s and iNOS in DSS mice were significantly decreased.

For *in vitro* study, RAW264.7 cells activated by LPS + IFN-γ were used for M1 polarization analysis. Our research showed that pretreatment with 1-MT inhibited LPS + IFN-γ-induced M1 polarization with significantly decreased levels of iNOS, TNF-α, and IL-6. By constructing IDO1 overexpression cell lines, we found that IDO1 overexpression alone drives macrophages towards M1 phenotype and raises inflammatory cytokines (TNF-α and IL-6). GRP78 and XBP1s expression levels differed significantly before and after IDO1 overexpression, positively correlating with IDO1 levels and were further increased upon LPS + IFN-γ induction. Subsequently, in vitro experiments revealed that inhibition of ER stress pathways using 4-PBA reversed M1 polarization induced by IDO1 overexpression in RAW 264.7 cell. Therefore, both *in vivo* and in vitro results strongly suggested that IDO1 might promote macrophage M1 polarization via ER stress-associated GRP78-XBP1 pathway.

Notably, within the tumor microenvironment, IDO1 facilitates the polarization of tumor-associated macrophages (TAMs) towards the immunosuppressive M2 phenotype through a metabolic cascade mechanism (49). This process involves the activation of the aryl hydrocarbon receptor (AhR)/STAT3 signaling axis by its metabolite kynurenine (KYN), subsequently inducing the expression of M2-associated markers including arginase-1 (Arg1) and IL-10. A parallel mechanism has been identified in hepatic fibrosis pathogenesis, where IDO1 establishes a pro-fibrogenic positive feedback loop between hepatic stellate cells (HSCs) and Kupffer cells through upregulation of TGF-β1/Smad3 signaling (50). This interaction potentiates M2 macrophage-mediated collagen deposition, with genetic ablation of IDO1 demonstrating remarkable efficacy by reducing fibrotic area by 60% in murine models. These findings present a partial discrepancy with our current conclusion regarding IDO1’s role in promoting M1 macrophage polarization.

In striking contrast, single-cell transcriptomic profiling reveals that IDO1-high macrophage subsets in Crohn’s disease exhibit marked enrichment of M1-associated signature genes (including iNOS, IL-6, and TNF-α), while demonstrating strong topological association with the development of mesenteric “creeping fat”—a hallmark pathological feature of intestinal inflammation (51). Moreover, ER stress emerges as a critical pathomechanism in IBD progression, functioning synergistically with IDO1-mediated immunometabolic reprogramming (23). These findings not only precisely corroborate our experimental conclusions but also provide mechanistic validation of the M1-polarization paradigm in chronic inflammatory disorders.

Collectively, these studies, along with our data, reveal a dichotomous regulatory role of IDO1 in macrophage polarization, acting as a double-edged sword in disease pathogenesis. It promotes immune-evasive M2 polarization in oncological and fibrotic contexts, yet paradoxically drives pro-inflammatory M1 polarization in IBD. This paradoxical duality may stem from organ-specific metabolic microenvironments that differentially activate downstream IDO1 signaling pathways, indicating a sophisticated context-dependent regulation of immunometabolic programming.

To advance our investigation into IDO1’s role in UC, future studies will incorporate established IDO1-knockdown murine models combined with systematic modulation of ER stress pathways. This will involve employing pharmacological modulators (4-PBA and other ER stress-related inhibitors/agonists) alongside genetic manipulation techniques targeting key ER stress components, ultimately enabling comprehensive elucidation of the precise molecular mechanisms through which IDO1 regulates ER stress responses in UC pathogenesis.

In addition, we are mindful of the limitations inherent in this study. The main limitation of this study was the small sample size of newly recruited UC patients. Although our *in vivo* and *in vitro* studies showed that IDO1 might modulate macrophage M1 polarization and inflammatory responses via the ER stress-associated GRP78-XBP1 pathway, whether similar effects occur clinically requires further validation.

## 5 Conclusion

Our research demonstrated that IDO1 interacted with the XBP1-GRP78 pathway related to ER stress to induce the transition of macrophage polarization towards the M1 phenotype, thereby facilitating the advancement of UC. Our discoveries are conducive to deepening the comprehension of the immune regulatory mechanisms of UC and offer a significant foundation for the development of novel therapeutic strategies.

## Data Availability

The datasets presented in this study can be found in online repositories. The names of the repository/repositories and accession number(s) can be found in the article/Supplementary material.

## Footnotes

^1^ https://cibersortx.stanford.edu/

## Supplementary material

SUPPLEMENTARY FIGURE 1 Analysis of myeloid cell subsets and related genes in healthy and inflamed colonic mucosa. **(A,B)** UMAP representation of scRNA-seq data for the myeloid cell subset proportions across healthy controls (HC, *n* = 6) and IBD colonic samples (CS *n* = 6, UC *n* = 6). **(C–E)** Co-expression analysis of IDO1, XBP1s, and GRP78.

SUPPLEMENTARY FIGURE 2 ELISA analysis of mouse colons. **(A)** The level of TNF-α in mouse colon tissue. **(B)** The level of IL-6 in mouse colon tissue. **p* < 0.05, ***p* < 0.01, ****p* < 0.001, and *****p* < 0.0001.
