# Supplementary figures and images for: IDO1 induced macrophage M1 polarization via ER stress-associated GRP78-XBP1 pathway to promote ulcerative colitis progression
Source: Front Med (Lausanne). 2025 Apr 30;12:1524952. doi: 10.3389/fmed.2025.1524952 (PMC12075526; doi:10.3389/fmed.2025.1524952)

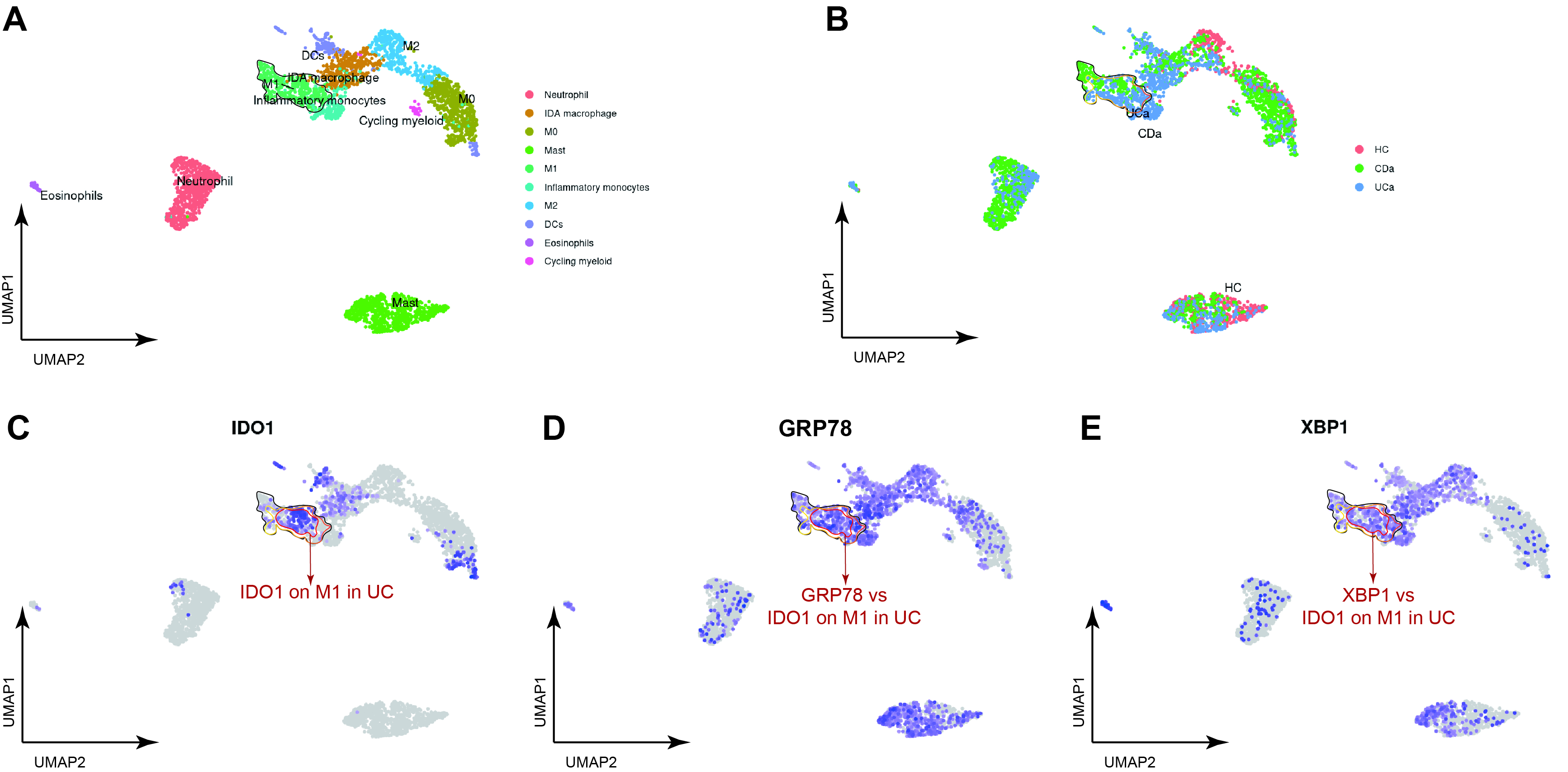

Supplement: SUPPLEMENTARY FIGURE 1 — Analysis of myeloid cell subsets and related genes in healthy and inflamed colonic mucosa. (A,B) UMAP representation of scRNA-seq data for the myeloid cell subset proportions across healthy controls (HC, n = 6) and IBD colonic samples (CS n = 6, UC n = 6). (C–E) Co-expression analysis of IDO1, XBP1s, and GRP78. [file Image_1.JPEG]

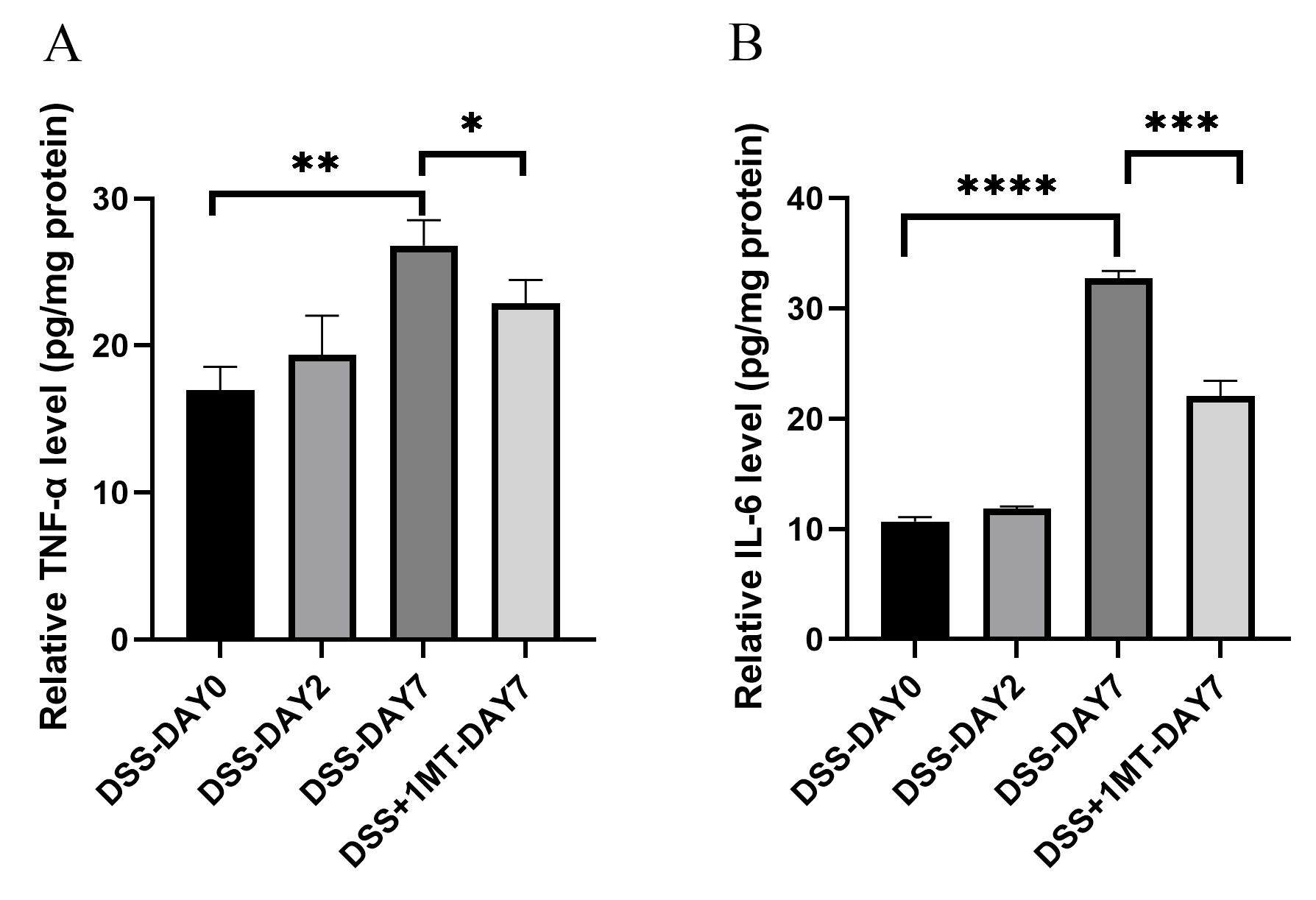

Supplement: SUPPLEMENTARY FIGURE 2 — ELISA analysis of mouse colons. (A) The level of TNF-α in mouse colon tissue. (B) The level of IL-6 in mouse colon tissue. *p < 0.05, **p < 0.01, ***p < 0.001, and ****p < 0.0001. [file Image_2.TIF]
